# Mimicking Complexity of Structured Data Matrix’s Information Content: Categorical Exploratory Data Analysis

**DOI:** 10.3390/e23050594

**Published:** 2021-05-11

**Authors:** Fushing Hsieh, Elizabeth P. Chou, Ting-Li Chen

**Affiliations:** 1 Department of Statistics, University of California at Davis, Davis, CA 95616, USA; 2 Department of Statistics, National Chengchi University, Taibei 116, Taiwan; eptchou@g.nccu.edu.tw; 3 Institute of Statistical Science, Academia Sinica, Taipei 115, Taiwan; tlchen@stat.sinica.edu.tw

**Keywords:** contingency-kD-lattice, high order structural dependency, heterogeneity, mutual conditional entropy matrix, principal component analysis (PCA)

## Abstract

We develop Categorical Exploratory Data Analysis (CEDA) with mimicking to explore and exhibit the complexity of information content that is contained within any data matrix: categorical, discrete, or continuous. Such complexity is shown through visible and explainable serial multiscale structural dependency with heterogeneity. CEDA is developed upon all features’ categorical nature via histogram and it is guided by all features’ associative patterns (order-2 dependence) in a mutual conditional entropy matrix. Higher-order structural dependency of k(≥3) features is exhibited through block patterns within heatmaps that are constructed by permuting contingency-*k*D-lattices of counts. By growing *k*, the resultant heatmap series contains global and large scales of structural dependency that constitute the data matrix’s information content. When involving continuous features, the principal component analysis (PCA) extracts fine-scale information content from each block in the final heatmap. Our mimicking protocol coherently simulates this heatmap series by preserving global-to-fine scales structural dependency. Upon every step of mimicking process, each accepted simulated heatmap is subject to constraints with respect to all of the reliable observed categorical patterns. For reliability and robustness in sciences, CEDA with mimicking enhances data visualization by revealing deterministic and stochastic structures within each scale-specific structural dependency. For inferences in Machine Learning (ML) and Statistics, it clarifies, upon which scales, which covariate feature-groups have major-vs.-minor predictive powers on response features. For the social justice of Artificial Intelligence (AI) products, it checks whether a data matrix incompletely prescribes the targeted system.

## 1. Introduction

Let a structured data set be represented by a rectangular matrix with sampled subjects being arranged along the row-axis and selected features arranged along the column-axis. Then, each entry of this data matrix is either a continuous or discrete feature’s measurement or a categorical feature’s category of a row-specific subject. This is the most common data structure encountered in data analysis. Naturally, we wonder: what information content is in such a data matrix? Interestingly, researchers seemingly never ask this simple and universally valid question formally in literature. Is it just like the most complex question taking the most simplistic form? “What is it?” John Steinbeck and his marine biologist friend E.D. Ricketts discussed and argued about the enormous hidden complexity in this simple question when he encountered the seemingly endless food-web in his 1951 book: “The Log from the Sea of Cortez” [1].

The wording of the above two questions points to the complex system that gives rise to the observed data set. Such a system could be rather commonly encountered in our real world. Even if it is entirely governed by well-understood physical and biological principles, its details always surprise us in one way or another. The more data we have, the more surprising structures and heterogeneity would merge upon finer and finer scales. This is a common phenomenon in the current Big Data era. When more data are collected from an intrinsically finite system of interest on this planet earth, its abundant structural scales would be exposed. Further, as we look into finer scales, the heterogeneous global and large-scaled structures would also become clear. This theme of multiscale structures with heterogeneity in any complex system was depicted in a 1972 Science paper title: “More is different” by physics Nobel laureate P. W. Anderson [2].

If multiscale structures with heterogeneity are underlying every finite complex system, should its data matrix’s information content not likewise embrace such structural characteristics? It is a solemn call for data analysts. Because our current system knowledge with available data analysis methodologies still cannot lead us to extract data’s full information content [3,4,5]. Such a state needs to be transcended.

Apparently, data analysts must adopt data-driven computing and effective exploration techniques to face the largeness of data. Their data analyzing repertoires must be widely expanded to discover complex multiscale structures and identify scale-specific heterogeneity. Such a transcendence could truly elevate Data Analysis as a discipline of science, as John Tukey argued and proclaimed in his 1962 paper with the title: “The future of Data Analysis” [6].

Data analysts and domain scientists share the central scientific issue: what makes this system of interest works as it does? Their scientific roles are somehow different. Domain scientists, in general, play the role of data curator, while data analysts do not. However, on the road to achieving an understanding of a target system, data analysts must check whether data curators indeed have achieved a somehow complete description of the target system or not? That is, data analysis should not begin by presuming that a complete description of the system of interest is in the data. This task already requires data analysts to successfully extract and discover the multiscale structures and identify the scale-specific heterogeneity contained in the data. Therefore, finding data’s full information content is not just the ultimate goal of data analysis, but also the most basic requirement. Only after this task is fulfilled, then making inferences become feasible and sensible.

**Figure 1:**
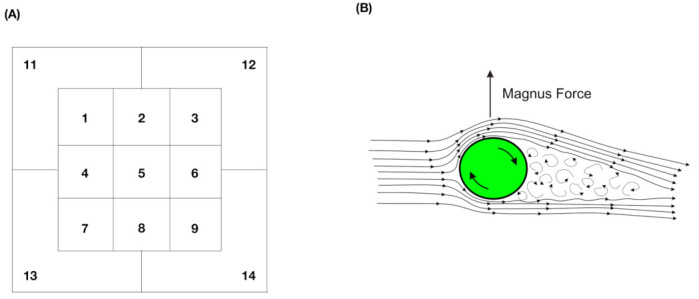
Two pictorial Illustrations: (**A**) strike “zone” in MLB; (**B**) Magnus effect for fastball having the back-spin and up-ward force, see https://upload.wikimedia.org/wikipedia/commons/I/15/Sketch_of_Magnus_effect (accessed on 8 May 2021).

We illustrate the above arguments with a real-world example. One rarely spoken fact is that data curators have intelligently encoded their expert knowledge and understanding regarding a system of interest into a data matrix. Such encoding is seen in the efforts of creating and choosing particularly relevant features. From this perspective, we see the significant differences between structured and unstructured data. For instance, PITCHf/x is a database from 2006 to 2017 seasons that was made public by Major League Baseball (MLB) in the USA. Two high-speed cameras take 40 images for every single pitch delivered by any MLB pitcher in any of 30 MLB stadiums. These images are unstructured data. To obtain structured data from these 40 images, scientists from the private company Sportvision have carefully calculated and meticulously selected 21 quantitative features. These features are created to capture each pitch’s bio-mechanical and aerodynamic characteristics. Several qualitative features, such as “batting_result”, “Strike _Ball”, and “zone _number”, see panel (A) of Figure 1, are also recorded for each pitch.

Specifically, we take a starting pitcher’s pitching dynamics of his favorite pitch-type, Four-seam Fastball, as the system of interest. On top of the aforementioned categorical features, features regarding his biomechanics are typically measured on the pitcher mound, while features regarding aerodynamics are measured along the baseball trajectory before arriving at the home plate. The most relevant physical rule within baseball pitching dynamics is called the Magnus effect in aerodynamics, as illustrated in panel (B) of Figure 1. Very briefly, the Magnus effect prescribes a force acting on the movement trajectory of a spinning subject, like a pitched baseball, within air media. The Fastball’s typical back-spin would induce an upward force as the Magnus effect, as illustrated in the panel. This is why a Fastball looks like going against gravity by flying upward when arriving at the home plate.

Here, the Magnus effect serves as one principal force in pitching dynamics [7]. Its chief factor is the baseball’s spin direction. There is only one feature, denoted as “spin_dir”, for spin-direction in PITCHf/x. Each discrete measurement of this feature is measured under the assumption that the Magnus effect is perpendicular to the baseball trajectory. However, a baseball field is wide and open, unlike a wind tunnel in a laboratory. Many minor forces could collectively impact a baseball trajectory. These minor forces are not measured and recorded in the data. For instance, there is one curve line of seams on each baseball’s surface, with which an MLB pitcher makes use of it differently to create distinct types of spins for his four-seam Fastball. One discrete measurement of “spin_dir” is unlikely to capture all of the seam’s rotating patterns. Further, a baseball trajectory is curved and more than 60 ft (18 m) long. From this trajectory perspective, this measurement of “spin_dir” is understood as just an approximated one. Furthermore, it is evident that atmospheric or air pressure, which is the key factor of the air media of a spinning baseball, would also have effects on the Magnus effect. However, it is left out. Accordingly, whether the PITCHf/x database indeed offers a complete or incomplete description of a pitcher’s pitching dynamics is an essential question to be addressed here. Such system completeness-vs.-incompleteness like questions should always be in data analysts’ minds.

As we attempt to understand a pitcher’s fastball pitching dynamics based on the PITCHf/x database, we have to simultaneously analyze a data matrix consisting of continuous, discrete, and categorical features. Such a structured data matrix of mixed data types is typical in the real world, like hospitals’ medical records, various censuses in governments, and business surveys. Given its prevalence in societies around us, it very mysteriously deems why we are by and large still missing efficient means to ascertain the whole and full information content contained in a data matrix with categorical entries.

Indeed, this phenomenon is not a mystery at all. Nowadays, in the literature of Mathematics, Statistics, or Computer Science, most methodologies that are popularly used for analyzing data purposes focus on sophisticated modeling and optimizations. These works universally require explicit Y=f(X) functional constructs to link data *Y* to data *X*. Nevertheless, there are no available functions that can naturally and efficiently accommodate categorical features at this data analysis stage. To transcend this current stage to accommodate reality, we need to go beyond modeling and its sole focus of inferences. To truly resolve fundamental issues in data analysis, we must extract any data matrix’s complete, or at least the essential parts of, information content. Here, it is emphasized that our data analysis must work equally well for all quantitative, all qualitative, or partly quantitative and partly qualitative data matrices.

What is a data matrix’s full information content looking like? It is not a formidable question. There are relatively simple, general settings in which we can visualize the total information content. A series of settings is given, as follows. If a data matrix has only one column corresponding to one feature, then its information content is seen as all patterns found in its histogram. A feature of any type universally has a histogram. Accordingly, we can see this data matrix’s information content. For a continuous feature, a very natural version of histogram, called a possibly-gapped histogram, is recommended [8]. This histogram accommodates a piecewise linear approximation to its empirical distribution function and potential gaps. They are major parts of data’s information content.

If there two features that are involved in a data matrix, then the data matrix’s information content ideally must add all associative patterns onto all patterns found on the two histograms, respectively. Here, associative patterns of two features of any data types can be seen through a contingency table framed by the two features’ histograms on row- and column-axes, respectively. A conditional categorical random variable is defined upon each row or column of this contingency table. These categorical random variables reveal a locality-specific directional associative relation, a visible kind of local heterogeneity. The strength of such directional associative relations can be evaluated by the Shannon entropy ratio between this categorical random variable and the marginal one. Row-wise and column-wise weighted sums of such Shannon entropy ratios, respectively, become a natural measure of the global directional associations, see the details in [9]. These local and global associative patterns are collectively representing the order-2 dependency of these two features.

When a data matrix involves three features, a 3D structure framed can be built using three histograms on three axes, respectively. Patterns of such a 3D structure collectively express the order-3 structural dependency among the features. However, visualizing and exploring such patterns is not an easy task. This task is impossible anyway for a data matrix with more than three features. Can we see the key characteristics of a complex high-dimensional relational structure if we pick and choose potential informative perspectives? Indeed, we can. We partially overcome this curse of dimensionality on human visualization by looking into K(≥3)D geometry through specific directions.

Here, we consider and propose taking a progressive approach given, as follows. Suppose that the three features are denoted as $\left\{X_{1},X_{2},X_{3}\right\}$ and assume that the pair $\left\{X_{1},X_{2}\right\}$ is most associative among the three feature-pairs. Subsequently, we have two choices: (1) make $\left\{X_{1},X_{2}\right\}$ as one whole; (2) split $\left\{X_{1},X_{2}\right\}$ and let $\left\{X_{2},X_{3}\right\}$ be one whole. A pair of features being taken as one whole means building a contingency table based on these two features for extracting this pair’s order-2 structural dependency. A larger contingency table, called the contingency-3D-lattice, is built with all of the occupied cells from the pair’s contingency table being arranged along the row-axis, while the bins of the histogram belonging to the third feature being arranged along the column-axis. For the first choice, its resultant contingency-3D-lattice provides a directional view of 3D structural dependency of $\left\{X_{1},X_{2},X_{3}\right\}$ from the perspective of $\left\{X_{1},X_{2}\right\}\Leftrightarrow X_{3}$, while the 2nd choice offers a directional view of 3D structural dependency from the perspective of $X_{1}\Leftrightarrow \left\{X_{2},X_{3}\right\}$. Indeed, these two choices carry rather distinct motivations. The perspective of $\left\{X_{1},X_{2}\right\}\Leftrightarrow X_{3}$ shows us what are new associative patterns between $\left\{X_{1},X_{2}\right\}$ and $X_{3}$. In contrast, the perspective of $X_{1}\Leftrightarrow \left\{X_{2},X_{3}\right\}$ demonstrates to us how and where $X_{3}$ would enhance the association of $\left\{X_{1},X_{2}\right\}$. The latter perspective is, as would be shown in sections below, one essential approach of visualizing heterogeneity in associative relations.

This contingency-3D-lattice in a large contingency table format might be too large to easily visualize pattern formations of associations and heterogeneity. Hence, we further proceed to build a heatmap based on this contingency-3D-lattice. A heatmap is resulted from properly permuting rows and columns respective to their natural neighborhood information. For a continuous feature or feature group, a tree is typically constructed from a binary adjacency matrix that records the natural neighborhood relations among all occupied cells or bins. Regarding categorical features, sometimes, a tree structure also could be established via domain knowledge.

Moreover, we need domain knowledge and natural neighborhood relations to permute their occupied cells for a feature-group of mixed types. Accordingly, a heatmap is built by superimposing a tree-geometry on the row-axis and another tree-geometry on the column-axis. The goal of permuting rows and columns of a contingency-3D-lattice into a heatmap is to reveal all possible block patterns. Under the tree-frameworks, such block patterns effectively capture the essential parts of order-3 structural dependency with evident heterogeneity. The most important characteristic of a heatmap is that the manifestation of deterministic and stochastic structures within such structural dependency becomes rather evident.

Regarding any data matrix involving K(>3) features of any data types, we likewise propose a step-by-step progressive split-then-aggregate protocol to discover essential high-order structural dependency of all involving features. That is, we build a series of contingency-*k*D-lattices and a corresponding series of heatmaps until all K(≥k) features are accommodated. In this serial fashion, we compute and visualize the multiscale structural dependency with heterogeneity as a major part of a data matrix’s information content. This serial multiscale structural dependency with heterogeneity would also become the basis for checking whether the targeted system has a complete or incomplete description given the set of chosen features.

A system of interest is typically defined by a designated set of response features and the rest of the features as covariate ones. Among covariate features, we can identify one of its subsets that collectively has major effects through directional conditional entropy evaluations along with the series of heatmaps. Further, we can also identify various subsets of features that only offer minor effects upon limited localities. Both major and minor effects together are sources of heterogeneity on each scale. The system’s complete or incomplete descriptions are realized along with identifying major and minor features. Consequently, we would see at which scale what response’ categorical structures can be predicted and what can not. All of the computational developments up to this point are called Categorical Exploratory Data Analysis (CEDA).

After CEDA, our mimicking protocol is proposed along with this series of contingency-*k*-lattices and the corresponding series of heatmaps, from the global to finest scales. Underlying this mimicking protocol is the principle of conditioning on observed deterministic and stochastic structural patterns with confirmed reliability [10]. That is, our mimicking protocol makes sure that each mimicry embraces the observable multiscale structural dependency and heterogeneity. Therefore, all reliability and robustness evaluations based on an ensemble of such mimicries, which can be huge, are coherent and pertinent without man-made assumptions or unrealistic perturbations on data’s computable and observable deterministic and stochastic structures. On top of these classic utilities, our mimicking can enhance our data visualization capability by filling up open spaces among observed data points in Euclidean space. With this advantage, we can see some intricate geometric structures of manifolds that are otherwise too blurred to be sure with only observed data points [11,12]. In this fashion, mimicking enhances the exposure of a data matrix’s information content complexity.

In Theoretical Information Theory, any data set has a conceptual Kolmogorov complexity. This complexity is referred to as the shortest computer program in a universal computer that can regenerate the data a one whole [13]. Although this is not a computable concept, it is highly relevant to most data analyses involving finding its pattern-based information content. In this paper, we take our mimicking as the flowchart of one computer program that is practically an approximation to the shortest one for Kolmogorov complexity.

This paper is organized, as follows. In Section 2, we discuss directional and mutual associations’ evaluations based on Shannon conditional entropy in detail. In Section 3 and Section 4, we discuss and develop CEDA with mimicking for all categorical and all continuous features settings, respectively. In Section 5, the mixed categorical and continuous setting is discussed. In Section 6, we discuss the impacts of CEDA with mimicking on Statistics, ML, and AI. Throughout this paper, our computational developments for CEDA and mimicking are fully illustrated with data matrices being constructed based on 2118 four-seam Fastball pitches delivered by the MLB pitcher: Justin Verlander of Houston Astro in the 2016 season. The data of these 2118 pitches are taken from MLB’s PITCHf/x database.

## 2. Multiscale Dependency and Directional Conditional Entropies

In this paper, a structured dataset is represented by a N×K data matrix that is denoted as $M_{0}=\left[X_{k}\left[i\right]\right]$. Its enrty $X_{k}\left[i\right]$ is either a categorical record, or a discrete or continuous measurement of *k*-th feature (or variable) $X_{k}$ from *i*th subject with i=1,…,N and k=1,…,K. These *K* features are possibly mixed in data types: continuous, discrete, or categorical. However, each feature of any type has its intrinsic categorical structure with respect to its histogram that is constructed based on the *N* 1D data points [8]. Within this collection ${\left\{X_{k}\right\}}_{k=1}^{K}$, at first, we do not separate response variables from covariate ones. We also consider no temporal or spatial coordinates within $M_{0}$.

### 2.1. Contingency-kD-Lattice

One basic construct in our CEDA developments is the contingency-*k*D-lattice. A contingency-1D-lattice is a vector of counts with respect to a histogram, while a contingency-2D-lattice is commonly called a contingency table, which is a matrix lattice of counts. Accordingly, a contingency-2D-lattice is framed by two contingency-1D-lattices with their occupied cells being arranged along its row- and column-axes, respectively. Hence, with $k=k_{1}+k_{2}$, a contingency-$k_{1}$D-lattice, and a contingency-$k_{2}$D-lattice can frame a contingency-*k*D-lattice by arranging their occupied cells along the row- and column-axes, respectively. As such, this contingency-*k*D-lattice would serve as one effective platform for viewing the order-*k* structural dependency of *k* features from a specifically chosen perspective between the feature-group of $k_{1}$ features and the feature-group of $k_{2}$ features.

A contingency-*k*D-lattice is a large contingency table. Underlying its largeness, heterogeneous structures likely are embedded. To make the difficult task of pattern recognition easier and simultaneously pave ways to reveal its explicit heterogeneity, we usually permute its row- and column-axes whenever possible. Here, the permutation performed on one axis typically means superimposing a hierarchical tree with all row-wise or column-wise occupied cells as tree-leaves. We intend to use a series of tree branches: from large to small, to cluster different degrees of neighboring occupied cells. When such a contingency-*k*D-lattice is framed by two such hierarchical trees, a multiscale of visible and explainable blocks would naturally appear. Therefore, a heatmap is a simple manifestation of embedded heterogeneity and large-scale order-*k* structural dependency with block-based deterministic and stochastic structures.

Here, we briefly discuss how to construct such a hierarchical tree under three settings. First, suppose that the $k_{1}$ features are all qualitative with nominal categories and $L_{1}$ occupied cells being found in contingency-$k_{1}$D-lattice. There exists no natural neighborhood system for these $L_{1}$ cells. However, some domain knowledge often helps build a hierarchical tree upon the $L_{1}$ cells in a realistic and meaningful fashion. Even without domain knowledge, we can treat each occupied cell as one population, and the contingency-*k*D-lattice becomes a setting of $L_{1}$ categorical sample problem, see [14]. A clustering tree would be available among $L_{1}$ cells. It is noted that qualitative features’ ordinal categories, indeed, are equipped with natural neighborhood systems.

Secondly, suppose that the $k_{2}$ features are all quantitative, and there are $L_{2}$ occupied cells found within the contingency-$k_{2}$D-lattice. With respect to the $R^{k_{2}}$ Euclidean geometry and a cell of contingency table being a $k_{2}$-dim $R^{k_{2}}$ hypercube, there are natural neighborhood systems among these $L_{2}$ hypercubes regarding their different kinds of connectivity: one point, one edge, or one face, etc. One chosen neighborhood system would give rise to one version of binary symmetric $L_{2}\times L_{2}$ adjacency matrix. One version of the adjacency matrix would specify one network structure among these $L_{2}$ cells. Therefore, various community-detection or clustering approaches, including the Hierarchical Clustering algorithm (HC), can be applied to build a hierarchical tree geometry upon the $L_{2}$ cells as nodes [15,16].

Thirdly, suppose that the $k_{3}$ features are of mixed types: quantitative and qualitative. In this setting, we recommend a bivariate-coding for these $L_{3}$ cells. Using domain knowledge to organize qualitative categories as alphabets {a,b,c,…} for the first coding-coordinate, and then quantitative categories as integers {1,2,3,…} as the second coding-coordinate, we arrange the $L_{3}$ cells on the column-axis in the following fashion: {a1,a2,…,b1,b2,…}. We will illustrate these three settings in Section 3 to Section 5, respectively.

### 2.2. Conditional Entropy and Order-k Structural Dependency in Contingency-kD-Lattices

If the number of features is *K*, around $2^{K}$ potential order-*k* structural dependency will be explored. Such full-scale explorations can easily overwhelm our computing capability. Further, because of our 3D visual limitation, we also need to devise a realistic approach to explore higher-order dependency when involving four or more features. For this reason, we employ contingency-*k*D-lattices as an extended contingency table embracing all *k*D hypercubes to resolve the visualization issue. The number of all potential contingency-*k*D-lattices could also overwhelm our computing capability. Accordingly, we need some kinds of road maps to guide us to see and pick potential essential ones. We attempt this task explicitly in this subsection.

As aforementioned, each feature of all data types has its intrinsic categorical nature. For a continuous $X_{k}$, it is categorized by a possibly-gapped histogram, which is computed via the Hierarchical Clustering (HC) algorithm [8]. Denote $X_{k}^{c}$ as the categorized version of $X_{k}$ and its categorical distributional nature via its histogram as $G_{k}$. In addition to its categorical nature, a discrete or continuous feature’s $G_{k}$ has a geometric neighbor system inherited from $R^{1}$. We also have $X_{k}^{c}=X_{k}$ when $X_{k}$ is categorical.

Within a contingency-*k*D-lattice, each unoccupied cell is a dependency structural pattern that indicates an impossible locality. The more unoccupied localities found in a contingency-*k*-lattice, the more structured dependency is revealed among the involved features. The structural dependency of order-*k* is easily seen via a locality-formation among empty cells and highly occupied cells. How to quantitatively evaluate such complex dependency structures in a 2D-table setting of any contingency-*k*D-lattice? We adopt several Shannon entropy-based elements from Combinatorial Information Theory, which are indeed model-free and scale-free. Such evaluations are equally applicable on heatmaps upon its hierarchical block-patterns formations.

Denote any pair of categorical or categorized features $\left\{X_{k}^{c},X_{k^{\prime }}^{c}\right\}$ with $k\neq k^{\prime }\in \left\{1,\ldots ,K\right\}$. Suppose that $G_{k}$ has *m* bins and $G_{k^{\prime }}$ has $m^{\prime }$ bins, respectively. All of the bins are indexed, as follows:$\left\{b_{j}|j=1,\ldots ,m\right\}$ for $X_{k}^{c}$, and $\left\{b_{j^{\prime }}^{\prime }|j^{\prime }=1,\ldots ,m^{\prime }\right\}$ for $X_{k^{\prime }}^{c}$. These two histograms $\left\{G_{k},G_{k^{\prime }}\right\}$ frame a $m\times m^{\prime }$ contingency table $Tab\left[m,m^{\prime }\right]=\left[n_{jj^{\prime }}\right]$ of counts $\left(n_{jj^{\prime }}\right)$ of subjects falling into 2D cells. Its row-sum vector is denoted by $\left(n_{1+},\ldots ,n_{m+}\right)$, and column-sum vector is denoted as $\left(n_{+1},\ldots ,n_{+m^{\prime }}\right)$. The total sum is $n_{++}=\sum_{j=1}^{m}n_{1+}=\sum_{j^{\prime }=1}^{m^{\prime }}n_{+1}=N$. This table would sustain two versions of directional conditional (re-scaled Shannon) entropy: column-to-wise ($X_{k}^{c}$-to-$X_{k^{\prime }}^{c}$) and row-wise ($X_{k^{\prime }}^{c}$-to-$X_{k}^{c}$), being denoted as $E[X_{k}^{c}|X_{k^{\prime }}^{c}]$ and $E[X_{k^{\prime }}^{c}|X_{k}^{c}]$. Below, we only detail the calculations for $E[X_{k}^{c}|X_{k^{\prime }}^{c}]$.

$$\begin{array}{ccc} E[X_{k}^{c}|X_{k^{\prime }}^{c}] & = & \frac{H[X_{k}^{c}|X_{k^{\prime }}^{c}]}{H[X_{k}^{c}]};\\ H[X_{k}^{c}|X_{k^{\prime }}^{c}] & = & \left(-1\right)\sum_{j^{\prime }=1}^{m^{\prime }}\frac{n_{+j^{\prime }}}{n_{++}}H\left[X_{k}^{c}|X_{k^{\prime }}^{c}=X_{k^{\prime },j^{\prime }}^{c}\right];\\ H[X_{k}^{c}|X_{k^{\prime }}^{c}=b_{j^{\prime }}^{\prime }] & = & \left(-1\right)\sum_{j=1}^{m}\frac{n_{jj^{\prime }}}{n_{+j^{\prime }}}\log\frac{n_{jj^{\prime }}}{n_{+j^{\prime }}};\\ H[X_{k}^{c}] & = & \left(-1\right)\sum_{j=1}^{m}\frac{n_{j+}}{n_{++}}\log\frac{n_{j+}}{n_{++}}. \end{array}$$

Here, $H[X_{k}^{c}]$ is the Shannon entropy of $X_{k}^{c}$, $H[X_{k}^{c}|X_{k^{\prime }}^{c}=b_{j^{\prime }}^{\prime }]$ is the conditional Shannon entropy of the conditional random variable $X_{k}^{c}$ given $X_{k^{\prime }}^{c}=b_{j^{\prime }}^{\prime }$, $H[X_{k}^{c}|X_{k^{\prime }}^{c}]$, the conditional Shannon entropy of $X_{k}^{c}$ given $X_{k^{\prime }}^{c}$, is calculated as a weighted sum of $H[X_{k}^{c}|X_{k^{\prime }}^{c}=b_{j^{\prime }}^{\prime }]$. The $E[X_{k}^{c}|X_{k^{\prime }}^{c}]$ is the re-scaled version column-wise direction conditional Shannon entropies. It is worth noting the following two facts that are related to the re-scaling used here.

1. If the the conditional entropy ratio $E\left[X_{k}^{c}|X_{k^{\prime }}^{c}=b_{j^{\prime }}^{\prime }\right]=\frac{H[X_{k}^{c}|X_{k^{\prime }}^{c}=b_{j^{\prime }}^{\prime }]}{H[X_{k}^{c}]}$ is indeed smaller than 1, then we know that the event $\left\{X_{k^{\prime }}^{c}=b_{j^{\prime }}^{\prime }\right\}$ in fact constrains and limits the potential outcomes of $X_{k}^{c}$. There might only be a small subset of $\left\{b_{j}|j=1,\ldots ,m\right\}$ that are coupled with $X_{k^{\prime }}^{c}=b_{j^{\prime }}^{\prime }$ being observed in $M_{0}$. This is the strong indication of directional association from one observed event of $X_{k^{\prime }}^{c}$ to $X_{k}^{c}$.
2. Because we have fact that $E[X_{k}^{c}|X_{k^{\prime }}^{c}]$ is the expected value or weighted average of $E[X_{k}^{c}|X_{k^{\prime }}^{c}=b_{j^{\prime }}^{\prime }]$. Hence, if $E[X_{k}^{c}|X_{k^{\prime }}^{c}]$ is indeed significantly smaller than 1, then, overall, the observed outcomes of $X_{k}^{c}$ are constrained and limited by outcomes of $X_{k^{\prime }}^{c}$. This is an overall directed association from $X_{k^{\prime }}^{c}$ to $X_{k}^{c}$ that allows us to make predictive results of $X_{k}^{c}$ based on outcomes of $X_{k^{\prime }}^{c}$.

Likewise, we can calculate directional conditional entropy $E[X_{k^{\prime }}^{c}|X_{k}^{c}]$ for the directional associations from $X_{k}^{c}$ to $X_{k^{\prime }}^{c}$. The simple average or minimum of these two directed conditional entropies is termed mutual conditional entropy (MCE) $E<k,k^{\prime }>$, see the details in [9]. The smaller the $E<k,k^{\prime }>$ value, the higher associative between $X_{k}^{c}$ and $X_{k^{\prime }}^{c}$. To avoid confusion, here we remark that our mutual conditional entropy $E<k,k^{\prime }>$ is intrinsically distinct with the commonly known mutual information ($I(X_{k}^{c}:X_{k^{\prime }}^{c})$). Their differences are seen, as follows.

$$\begin{array}{ccc} E<k,k^{\prime }> & = & [E\left[X_{k}^{c}|X_{k^{\prime }}^{c}\right]+E\left[X_{k^{\prime }}^{c}|X_{k}^{c}\right]]/2,\mathrm{or}\\ & = & \min E\left[X_{k}^{c}|X_{k^{\prime }}^{c}\right],E\left[X_{k^{\prime }}^{c}|X_{k}^{c}\right],\\ I(X_{k}^{c}:X_{k^{\prime }}^{c}) & = & H\left(X_{k}^{c}\right)-H\left(X_{k}^{c}|X_{k^{\prime }}^{c}\right),\\ & = & H\left(X_{k}^{c}\right)+H\left(X_{k^{\prime }}^{c}\right)-H\left(X_{k}^{c},X_{k^{\prime }}^{c}\right). \end{array}$$

Thus, $I(X_{k}^{c}:X_{k^{\prime }}^{c})$ is a difference of two quantities without re-scaling, while $E<k,k^{\prime }>$ is properly re-scaled. The scaling makes $E<k,k^{\prime }>$ to reflect the rate-comparisons between conditional and marginal entropies pertinently. The value “1” becomes a standard with a special meaning. However, mutual information $I(X_{k}^{c}:X_{k^{\prime }}^{c})$ has no reflecting standards. That is why MCE becomes a sensible association measure for two variables.

Based on the collection of histograms ${\left\{G_{k}\right\}}_{k=1}^{K}$, we compute all of the pairwise MCEs to make a K×K mutual conditional entropy (MCE) matrix, being denoted as $M_{ce}=\left[E<k,k^{\prime }>\right]$. Further, if we take the degree of association as a form of closeness, then $M_{ce}$ can be taken as distance matrix among ${\left\{X_{k}^{c}\right\}}_{k=1}^{K}$. We apply the Hierarchical clustering (HC) algorithm on $M_{ce}$ and build a binary HC-tree $T_{mce}$.

The heatmap $M_{ce}^{T}$ of $M_{ce}$ is obtained by superimposing tree $T_{mce}$ onto its row and column axes. Here, by superimposing, we mean the permuting operations with respect to the arrangement of tree-leaves of $T_{mce}$. Accordingly, upon the heatmap $M_{ce}^{T}$, we would see block-patterns displayed along the diagonal of the matrix lattice. Because the tree geometry has multiple levels, such block-patterns are also multi-scale. Upon each block of choice, it corresponds to a highly associative or so-called synergistic feature-group. That is, the heatmap $M_{ce}^{T}$ can serve as a road map that indicates which features are highly associated with which features, but less associated with which features. Algorithm 1 provides the procedure to build the heatmap.

After the above Combinatorial Information Theory discussion and developments, from here onward to the end of this paper, we would not explicitly differentiate between $X_{k}$ and its categorized version $X_{k}^{c}$. It is just for notational simplicity. The context should make clear which versions are indeed used without specific indications. For instance, all of the entropies of $X_{k}$ are always evaluated with respect to $X_{k}^{c}$. We do not consider any entropies of any continuous feature or random variable in this paper.
**Algorithm 1:** Heatmap based on mutual conditional entropy**Input**: N×K data matrix $M_{0}=\left[X_{k}\left[i\right]\right]$.
**Output**: The heatmap $M_{ce}^{T}$.
For each pair *k* and $k^{\prime }$, compute $E\left[X_{k}^{c}|X_{k^{\prime }}^{c}\right]=\frac{H[X_{k}^{c}|X_{k^{\prime }}^{c}]}{H[X_{k}^{c}]}$.
The K×K mutual conditional entropy matrix is defined as $M_{ce}=\left[E<k,k^{\prime }>\right]$, 
where $E<k,k^{\prime }>=\left[E\left[X_{k}^{c}|X_{k^{\prime }}^{c}\right]+E\left[X_{k^{\prime }}^{c}|X_{k}^{c}\right]\right]/2$.
Apply Hierarchical clustering algorithm on $M_{ce}$ to build a binary HC-tree $T_{mce}$.
Permute the rows and columns of $M_{ce}$ according to the arrangement of tree-leaves of $T_{mce}$ to have $M_{ce}^{T}$. 


**Figure 2:**
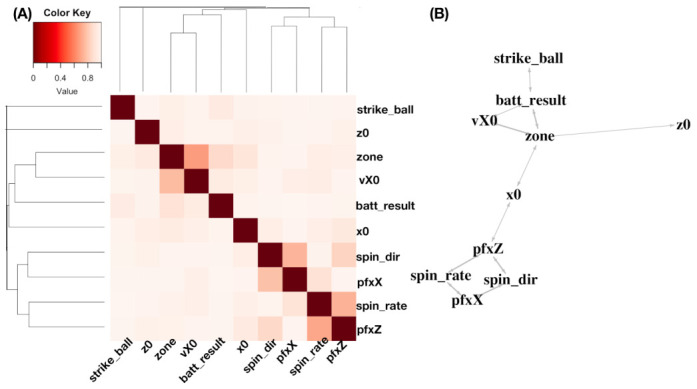
(**A**) Heatmap of 10×10 MCE matrix (with a HC tree using the single linkage module) and (**B**) a directed network of 10 nodes with thin and thick edges for directional conditional entropy falling in threshold regions [0.9,0.95] and [0,0.9], respectively.

We illustrate the heatmap $M_{ce}^{T}$ based on a 2118×10 data matrix $M_{0}$, as shown in panel (A) of Figure 2. There are three synergistic feature-groups: 1) {“pfx_x”(*Y*), “spin_dir”(*X*), “pfx_z”(*V*), “spin_rate”(*U*)} for Magnus effect; 2){“VX0”, “X0”, “Z0”} for the biomechanics of pitching gesture; 3){“Zone”, “Batting-results”, “Strike-vs.-Ball”}. Upon the point-clouds in $R^{4}$ and $R^{3}$ of the first and second synergistic feature-groups and rotatable figures of these manifolds that are presented in [17], respectively. Directional conditional entropies of $E[X_{k^{\prime }}^{c}|X_{k}^{c}]$ among the 10 feature-nodes beyond a chosen threshold are also presented as directed linkages in panel (A) of Figure 2.

## 3. CEDA with Mimicking Scenario-I: All Categorical Features

We first develop our CEDA paradigm on categorical data matrix setting in this section. All of the computational developments are fully illustrated through a real 2118×3 data matrix consisting of all four-seam fastball pitches delivered by MLB pitcher Justin Verlander in the 2016 season. Three categorical features: {“Zone”, “Batting-results”, “Ball-vs.-Strike”}. There are 12 count-categories in the format of a−b: *a* Balls and *b* Strikes, 11 batting-result categories, and 13 zones: nine strike zones and four peripheral zones outside of strike zone. Their 3×3 mutual conditional entropy (MCE) matrix can be extracted from panel(A) of Figure 2. The pair of two features—“Zone”, “Batting-results”—is slightly more associative (with directional conditional entropies 0.85 for the zone-to-batting result and 0.89 for batting-result-to-zone) than the other two pairs of features (with MCEs being equal to 0.940 and 0.961). It is recalled, from the Introduction section, that we would split this most associative pair when we construct the contingency-3D-lattice below.

[Discover order-2 structural dependency for mimicking: 1st step.]

**Figure 3:**
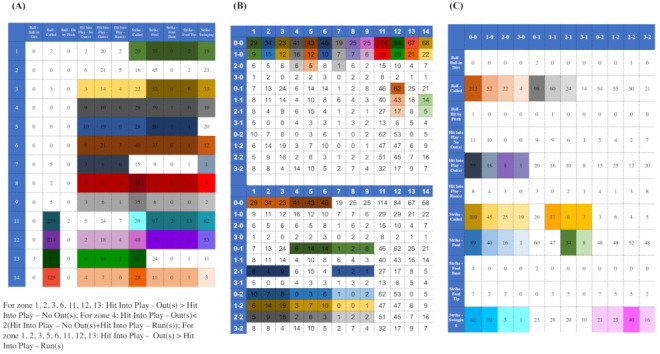
Contingency tables marked with constraints with 95% reliability: (**A**) Zone-vs.-Batting-results; (**B**) Strike-and-Ball-vs. Zone; and, (**C**) Batting-results-vs.-Strike-and-Ball. (the darker color, the higher order.)

In this example, there are three pairs of categorical features. We look for evident patterns from all three corresponding contingency tables (contingency-2D-lattices). All of the patterns are picked up either based on observed evidence or domain knowledge. Each pattern is subject to the reliability check. For this reliability check, a large ensemble of simulated sets of 2118 copies of 3D vectors that are generated by a simple random sampling scheme. A pattern is only turned into a rule of regulating purpose (under our would-be proposed mimicking protocol) when 95% of the members of the ensemble retain this pattern. The collection of rules with reliability are to regulate our first step of mimicking collectively. These rules are marked on the three contingency tables shown in Figure 3. They are visible and explainable.

Indeed, they represent 2D structural dependency within each of the three contingency tables. That is, we can see which feature has predictive power for which feature on which scale. For example, panel (A) shown in Figure 3, the category of Ball-call (2nd column), can predict rather well for the four peripheral zones: {zone-11, zone-12, zone-13, zone-14}, as one composite category, but not individual zones. Such kinds of feature-sensitive and scale-specific aspects of information content include: being good at a specific feature’s certain composite subset of categories, but not individual ones, are seen among all the three contingency tables. This might be the reality of data’s information content in the real world. The next question is: can the “Zone” be better predicted by using {“Batting-results”, “Ball-vs.-Strike”}?

In this step of discovering order-2 structural dependency, it is critical to mention that, in our experiment, we found that the collective acceptance rate is less than 35% within the aforementioned large ensemble of simulated sets of 2118 copies of 3D categorical vectors. That is, the rejection rate is more than 65%. This is one rather significant different as compared to other re-sampling schemes, such as Bootstrapping [18].

[Construct a contingency-3D-lattice for mimicking: 2nd step.]

After discovering the order-2 structural dependency, we proceed to explore and discover the order-3 dependency among the three categorical features: {“Zone”, “Batting-results”, and “Ball-vs.-Strike”}. This series of structural dependency forms the basis of information in the 2118×3 data matrix. The order-3 structural dependency surely would help us to address the aforementioned natural question: can “Zone” be better predicted by using {“Batting-results” and “Ball-vs.-Strike”}?

To capture order-3 structural dependency of these three features and shed light on the above question, we construct a contingency-3D-lattice of “Zone” vs. {“Batting-results”, “Ball-vs.-Strike”}. There are 13 categories of “Zone” (without zone-10) and 95 occupied cells in the contingency table of {“Batting-results”, “Ball-vs.-Strike”}, as shown in panel (C) of Figure 3. Therefore, we have a 13×95 a contingency-3D-lattice with all of its rows and columns being categorical.

**Figure 4:**
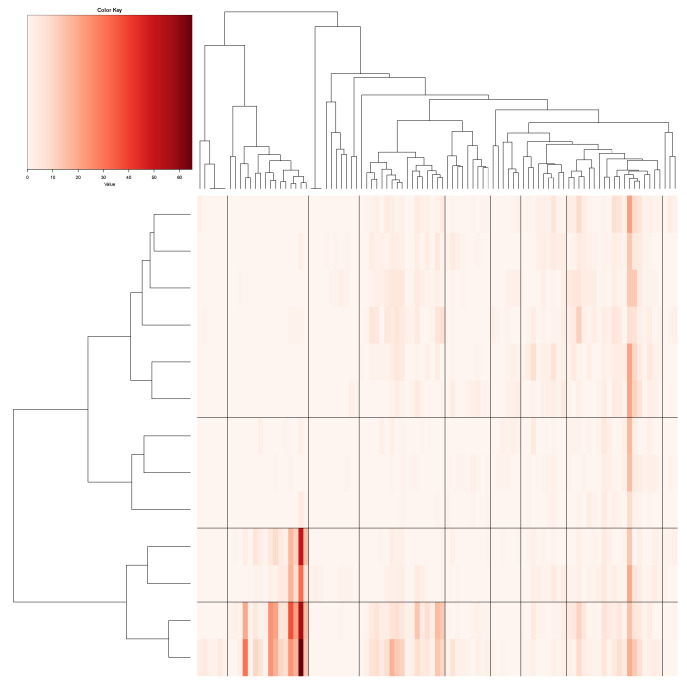
The 13×95 contingency table of the zone feature against the bivariate feature Ball-vs.-Strike count and Batting result.

To transform this contingency-3D-lattice into a heatmap, each row vector is considered to be a 95-dim vector of categorical counts pertaining to one population. This vector can be turned into a proportion vector by dividing by its row sum. Then we have a data set consisting of 13 populations, from each of which a proportion vector of 95-dim is observed. This is a typical Extreme-*K* categorical sample problem that is equipped with natural distance measures, see the details in [14]. An HC-tree is derived upon these 13 rows. Likewise, we have another Extreme-*K* categorical sample problem with 95 populations, each of which has an observed 13-dim vector of proportions. Accordingly, another HC-tree is derived. By superimposing these two HC-trees on the row and column axes, respectively, we arrive at a heatmap marked with 4 lattice of blocks, as shown in Figure 4. The visible and explainable patterns that are revealed by this heatmap are an order-3 structural dependency of these three categorical features. We acknowledge that such a collection of patterns is an essential part of the information content contained in the 2118×3 observed data matrix. Algorithm 2 summarizes the algorithm for building the contingency-*k*D-lattice.
**Algorithm 2:** Building the contingency-*k*D-lattice**Input**: A contingency-$k_{1}$D-lattice and a contingency-$k_{2}$D-lattice. **Output**: The contingency-*k*D-lattice.
If $k_{1}>1$, treat each occupied cell in the contingency-$k_{1}$D-lattice as a new category. 
If $k_{2}>1$, treat each occupied cell in the contingency-$k_{2}$D-lattice as a new category. 
Construct the contingency-*k*D-lattice with each row representing an occupied category from the contingency-$k_{1}$D-lattice and each column representing an occupied category from the contingency-$k_{2}$D-lattice. 
Permute the rows and columns, respectively, by the HC-trees from [14]. 


A cluster that was found on the row-axis would indicate that its member categories share the same functional relations of having close conditional distributions with respect to the categories and clusters on the column-axis and vice versa from the opposite direction. For instance, there are four evident clusters of zone-numbers on row-axis (from top to bottom): {1,2,3,4,5,6} (the upper part of strike zone); {7,8,9} (lower part of strike zone); {13,14} (lower peripheral of strike zone); and, {11,12} (upper peripheral of strike zone). Moreover, there are nine evident clusters along the column-axis. These clusters on both axes together construct a block framework as the large-scale order-3 structural dependency. We summarize and explain this visible block-patterns, as follows.

1. The four zone-specific clusters are rather heterogeneously distributed across the clusters of bivariate categories of {“Batting-results” and “Ball-vs.-Strike”}. Further, these four block-row specific heterogeneity are distinct, so are their relational functions.
2. The clear separation between block-rows {13,14} and {11,12} across column-clusters of {“Batting-results” and Ball-vs.-Strike”} belonging to the left-major branch, but not clusters belonging to the right-major branch, reveal apparent order-3 structural dependency.
3. The apparent heterogeneity that is revealed on this heatmap in Figure 4 indicates that “Batting-results” and “Ball-vs.-Strike”, together, play the minor effect role only. Therefore, the answer to the above question is that “Ball-vs.-Strike” only helps “Batting-results” upon some limited numbers of localities, not uniformly.
4. The order-3 structural dependency manifested through the 4 lattice of blocks in this 13×95 heatmap offers functionally meaningful information of clustering among the 2118 pitches.

[Mimicking protocol for scenario-I.]

Let’s take the series: the 11×12 contingency-2D-lattice of {“Batting-results”, “Ball-vs.-Strike”}, as shown in panel (C) of Figure 3, and the 13×95 contingency-3D-lattice of “Zone” vs. {“Batting-results”, “Ball-vs.-Strike”}, as shown in Figure 4, as the basis for our mimicking protocol given as follows.

**M1-1**. We first simulate a 11×12 contingency-2D-lattice of {“Batting-results”, “Ball-vs.-Strike”} that satisfies all of the rules marked there;
**M1-2**. The 95 simulated counts from contingency-2D-lattice in Step-[M1-1] are taken as the 95 column sums for column-by-column simulations for a 13×95 contingency-3D-lattice of “Zone” vs. {“Batting-results”, ”Ball-vs.-Strike”}.
**M1-3**. A simulated 13×95 contingency-3D-lattice in Step-[M1-2] is accepted if the resultant contingency tables of {“Zone”,“Batting-results”} and {“Zone”, “Ball-vs.-Strike”} satisfy all of the rules marked in panel (A) and (B) of Figure 3.

Algorithm 3 describes the generic algorithmic flow-chart of this protocol.
**Algorithm 3:** Micmicking protocol for contingency-*k*D-lattice for scenario-I
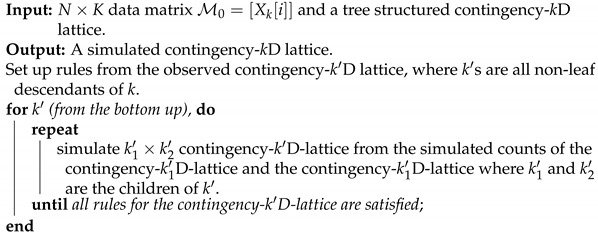


This mimicking protocol makes sure that each mimicry embraces all of the computed pattern information with reliability from the observed data matrix. It is noted that an ensemble of mimicries can be of any size. Such an ensemble would be the proper basis for evaluating any computational approaches that are applied on this 2118×3 data matrix, such as clustering structures among the 2118 pitches and its robustness. It is emphasized once more that our mimicking might give rather distinct evaluations from that of classic approach.

In this section, we demonstrate the theme of this paper under the scenario of all categorical features: our CEDA computations extract the observed data matrix’s multiscale information content, which is manifested through a series of various orders of structural dependency with heterogeneity, and our mimicking protocol is based on this information content.

## 4. CEDA with Mimicking Scenario-II: All Continuous Features

In this section, we consider mimicking a N×K data matrix with all *K* features of continuous measurements. These features are again denoted as ${\left\{X_{k}\right\}}_{k=1}^{K}$ with K>1. Upon each feature, one histogram is built and used as the basis for “categorizing” this continuous feature. Based on the categorical nature of these *K* categorized features, we compute K×K the MCE matrix as a road map for detecting 2D dependency for all feature-pairs. As for discovering high orders dependency beyond that of order-2, we would employ contingency-*k*D-lattice in the format of “2-to-1” for order-3, and ”3-to-1” and “2-to-2” for order-4, etc. We specifically consider a scenario of having a two-layer structured dependency to illustrate our CEDA with mimicking computational developments here. The *K* features are subdivided into two synergistic feature-groups, and these groups are globally associated.

To concretely illustrate such a two-layer structured dependency scenario, we use a feature-group of K=4: {“pfx_x”, “spin_dir”, “pfx_z”, and “spin_rate”}. This feature-group primarily manifests the Magnus effects in aerodynamics of baseball pitching, as illustrated in panel (B) of Figure 1. These four features consists of two synergistic pairs: {“pfx_x”, “spin_dir”} and {“pfx_z”, “spin_rate”}. Their within-pair associations are larger than their between-pair association, as shown in Figure 2. Such a two-layer structural dependency allows us to illustrate the protocols for mimicking based on two contingency-2D-lattices in the format of “1-to-1”, and then a contingency-4D-lattice in a format of “2-to-2”. This mimicking protocol can be easily modified to accommodating various formats, like“2-to-1” and “3-to-1”.

**Figure 5:**
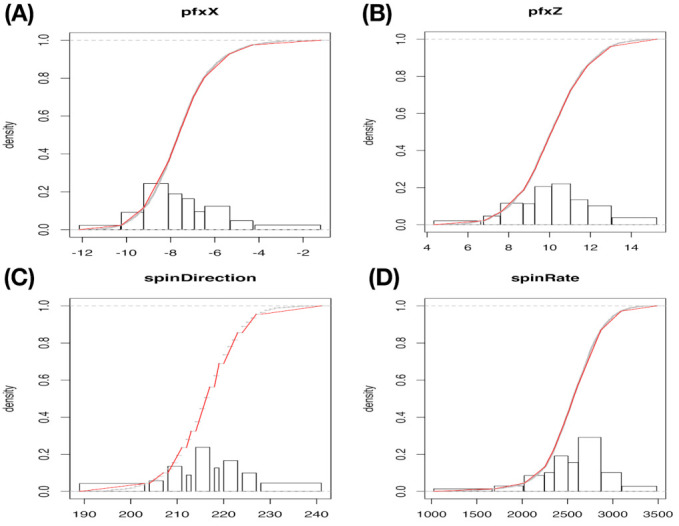
Piecewise linear approximations via histograms of four features related to Magnus effects: (**A**) “pfx_x”; (**B**) “pfx_z”; (**C**) “spin_dir”; and, (**D**) “spin_rate”.

Recall that the actual N×4 data matrix used here is derived from the same collection of 2118(=N) pitches of the four-seam Fastball delivered by MLB pitcher Justin Verlander in the Year 2016 season. The individual categorical nature of these four features are revealed through their own histogram that is reported in Figure 5, and the degrees of validity of histograms are demonstrated by giving rise to very well piecewise linear approximations to their empirical distributions.

**Figure 6:**
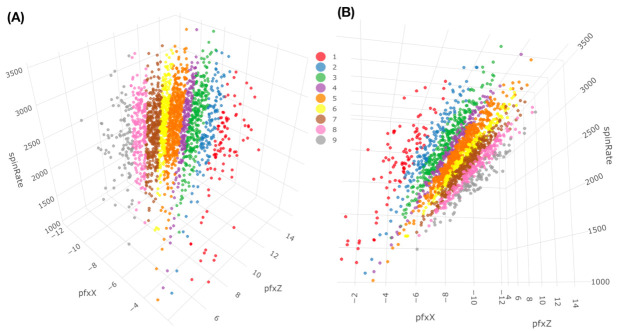
The 4D manifold of 4 features related to Magnus effects. The color-coding scheme is the 9 bins of histogram of “spin_dir”: (**A**) front view; (**B**) back view. See corresponding rotatable 3D plots through the link: https://rpubs.com/CEDA/Mimicking (accessed on 8 May 2021).

If we use a color-coding scheme based on nine bins in the histogram of “spin_dir”, then the 4D geometry of these 2118 data points indeed can be expressed by a colored 3D manifold as shown in Figure 6. As shown via the two panels, the front and back views of this colored 3D manifold reveal the nearly 2D geometric structures. Indeeds, such a low complexity structure manifests the Magnus effect by indicating highly structural dependency among these four features.

### 4.1. Multiscale Structural Dependency

**Figure 7:**
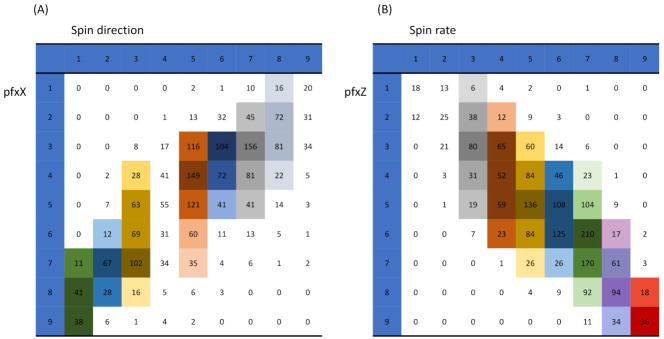
Two 9×9 contingency tables marked with 95% pattern-based rules: (**A**) {“pfx_x”, “spin_dir”}; (**B**) {“pfx_z”, “spin_rate”}. Pattern-based rules are marked along columns: darker colors for larger counts.

For visualizing the structural dependency of these two highly associated feature-pairs, {“pfx_x”, “spin_dir”}, and { “pfx_z”, “spin_rate”}, we make use of their 9×9 contingency tables as the simplest natural platform, as shown in Figure 7. Both of the contingency tables, in fact, effectively bring out the geometric patterns of dependency in a visible and explainable fashion. We see evident column-wise unimodal patterns of counts evolving along the diagonals of both tables. Likewise, unimodal patterns are also visible along the row-axis, but with slightly fewer degrees.

Are these unimodal patterns realistic in the sense of being supported by data? We go through the same reliability checking protocol as if we are under the categorical setting. For expositional simplicity, we focus on column-wise patterns and report the 95% reliable rules, as marked in Figure 7. These are rules that would be implemented when we perform the mimicking protocol below.

Before discussing our mimicking protocol, we need to overcome one obvious issue: many cells in both contingency tables have rather small counts. We need to resolve such an issue by merging. Because each data point is 4D, we need to use data’s 4D geometric information and their structural dependency. We propose using the neighborhood systems of two collections of occupied cells in both contingency tables to resolve this issue. There are 62 occupied cells for the contingency table of {“pfx_x”, “spin_dir”}, and 53 for that of {“pfx_z”, “spin_rate”}.

By a neighborhood system of a collection of 62 cells, we mean a binary symmetric 62×62 adjacency matrix that records which cells are neighbors of which cells. There are two natural definitions of the neighbor of two cells: (1) sharing a common edge; and, (2) sharing a common edge or a point of a corner. A cell can have four neighbors under the first definition but can have 8 neighbors under the second definition. An adjacency matrix based on the four-neighbor definition would give rise to a tighter relational neighborhood than the eight-neighbor definition. The visible consequence is that many clusters with a small number of member cells, but tight connectivity, would result from the four-neighbor definition. Accordingly, this neighbor definition is suitable for a large data set. In contrast, there would be considerably fewer clusters with a sizable number of member cells but loose connectivity, from the eight-neighbor definition. Therefore, this definition is suitable for contingency tables with many sparsely occupied cells, as we have seen in this case.

Here, we apply the Hierarchical clustering (HC) algorithm to build a HC-tree on the two binary symmetric 62×62 and 53×53 adjacency matrices for {“pfx_x”, “spin_dir”}, and {“pfx_z”, “spin_rate”}, respectively. To further reveal potentially more detailed global dependency structures, we build a 62×53 contingency table that is based on the two collections of occupied cells from both contingency tables. There are 293 occupied cells among the total 3286(=62×53 cells in the contingency table. That is, the fact of having only 8.9% of cells being occupied strongly indicates the structural dependency between {“pfx_x”, “spin_dir”} and {“pfx_z”, “spin_rate”} on fine-scale. Each occupied cell of this 62×53 contingency table is a 4D hypercube that is framed by foue bins of the four features’ histograms.

**Figure 8:**
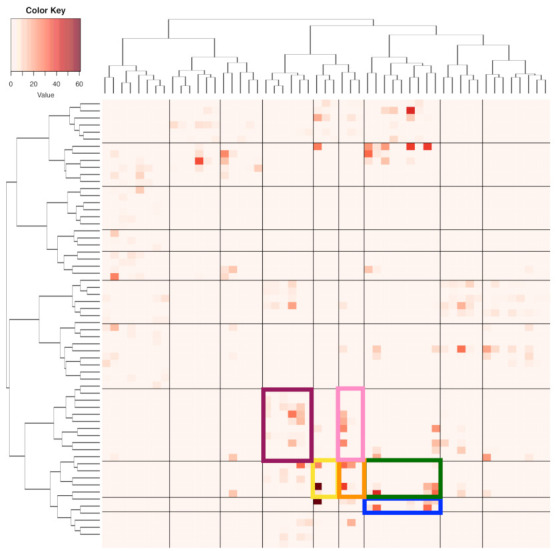
Contingency-4D-lattice of 4 features governing Magnus effects: { “pfx_x”, “spin_dir” }-vs.- { “pfx_z”, “spin_rate”}.

Next, we make such a fine-scale version of structural dependency visible and, at the same time, look for large-scale versions. To achieve both tasks, we superimpose the two neighborhood-system-based HC-trees accordingly on the row-and column-axes to result in a heatmap, as shown in Figure 8. The large-scale versions of the four features’ structural dependency are explicitly expressed through the multiscale block-structures that are framed by a composition of clusters on row-axis and another composition of clusters on column-axis found on the heatmap of Figure 8. Hence, one collection of occupied blocks could serve as a version of a large-scale structural dependency. We choose one version of such block-based structural dependency to serve as the foundation for mimicking the observed 2118×4 data matrix. This choice is primarily made to ensure that there is only a small number of blocks containing less than 10 data points. It turns out that there are 10 blocks found in the block-composition that is shown in Figure 8 having less than 10 data points. These blocks would be kept intact in our mimicking protocol because of the high potentials of unreliable structural geometry.

### 4.2. Mimicking Based on One Block

A chosen block within the heatmap, such as one of the six colored blocks shown in Figure 8, typically consists of a small collection of adjacent 4D hypercubes of {“pfx_x”, “spin_dir”, “pfx_z”, and “spin_rate”}. These 4D hypercubes likely have relatively uniform 4D geometric characteristics due to being adjacent to each other. Therefore, such a block can be taken as a super-4D-hypercube.

**Figure 9:**
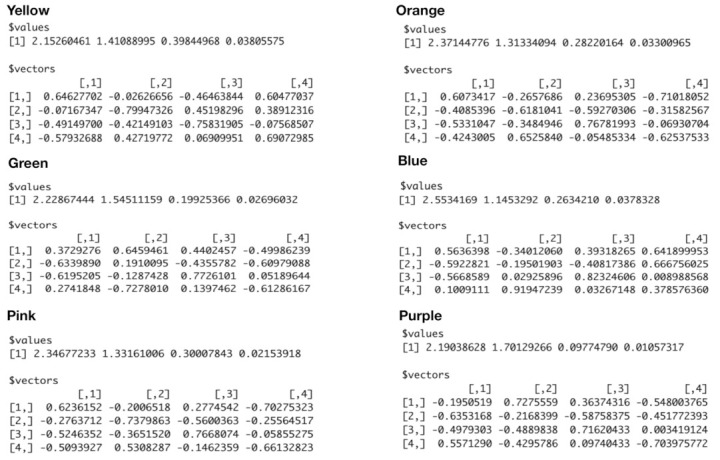
Eigenvalues and eigenvectors form six color-coded blocks.

**Figure 10:**
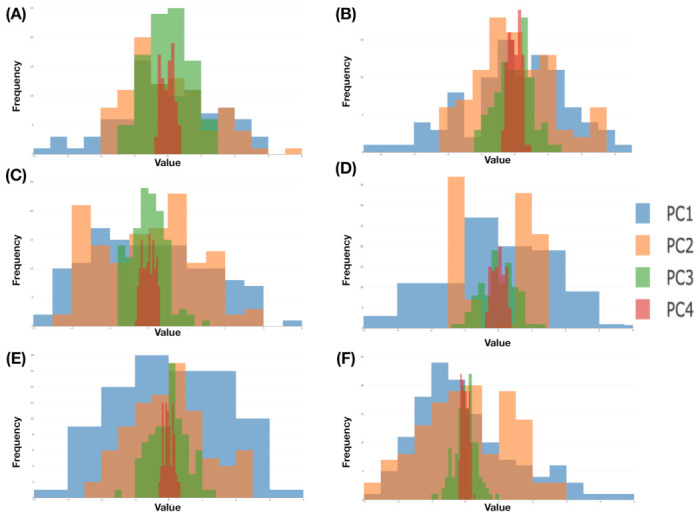
Individual histograms of ${\tilde{\varepsilon }}_{i}^{\left(1\right)},{\tilde{\varepsilon }}_{i}^{\left(2\right)},{\tilde{\varepsilon }}_{i}^{\left(3\right)},{\tilde{\varepsilon }}_{i}^{\left(4\right)}$ pertaining to the 6 color-coded blocks: (**A**) Yellow; (**B**) Orange; (**C**) Green; (**D**) Blue; (**E**) Pink; and, (**F**) Purple.

Denote the measurements of these four features as {X,U,Z,W} with {X,U} for {“pfx_x”, “spin_dir”} and {Z,W} for {“pfx_z”, :spin_rate”}, and denote the observed 4D point-cloud contained in this block (□) as an ensemble $C_{□}^{o}={\left\{C_{i}^{o}={\left(x_{i},u_{i},z_{i},w_{i}\right)}^{T}\right\}}_{i=1}^{m}$ with *m* copies of 4×1 vectors of observed measurements. We perform Principal Component Analysis (PCA) upon the m×4 data matrix and then extract the four eigenvalues $\left(\lambda_{1},\lambda_{2},\lambda_{3},\lambda_{4}\right)$, which are arranged in decreasing fashion, and their corresponding four eigenvectors $\left\{V_{1},V_{2},V_{3},V_{4}\right\}$. Here, $V_{h}$ is in a 4×1 format and its transpose is denoted as $V_{h}^{T}$ with h=1,2,3,4. We expect that the smallest eigenvalue $\lambda_{4}$ would be rather small as being close to 0, while the third eigenvalue $\lambda_{3}$ could be only slightly larger than $\lambda_{4}$, as seen in Figure 6. These two observations are confirmed below. Further, we denote the 4×4 matrix $V_{□}=\left[V_{1}^{T}:V_{2}^{T}:V_{3}^{T}:V_{4}^{T}\right]$ by stacking the four 1×4 transposed eigenvectors as four row vectors. Additionallt, projections of $C_{i}^{o}$ onto the coordinate system with respect to the four eigenvectors are then denoted and calculated as:

$$\varepsilon \left[i|□\right]=V_{□}C_{i}^{o},$$

with $\varepsilon \left[i|□\right]={\left(\varepsilon_{i}^{\left(1\right)},\varepsilon_{i}^{\left(2\right)},\varepsilon_{i}^{\left(3\right)},\varepsilon_{i}^{\left(4\right)}\right)}^{T}$ and i=1,...,m. We then build four histograms $H^{\left(h\right)}$ with h=1,..,4, each of which is based on ${\left\{\varepsilon_{i}^{\left(h\right)}\right\}}_{i=1}^{m}$. We illustrate the aforementioned computational results derived from six color-coded blocks that are shown in Figure 8. The six eigenvalues and eigenvectors are reported in Figure 9, while the six histograms are reported in Figure 10, respectively.

It is evident that ranges of ${\tilde{\varepsilon }}_{i}^{\left(1\right)},{\tilde{\varepsilon }}_{i}^{\left(2\right)},{\tilde{\varepsilon }}_{i}^{\left(3\right)}$ and ${\tilde{\varepsilon }}_{i}^{\left(4\right)}$ are rather distinct. This observation supports our visualization of a “nearly” 2D manifold of 4D measurements of the four Magnus effect-related features. It also potentially indicates that structural details could be rather intricate in one whole, on the other hand. Indeed, to realize such potential visually is the most immediate impact of mimicking.

By beginning with mimicking within a block, we build their kernel smooth versions, denoted as ${\tilde{H}}^{\left(h\right)}$ with h=1,…,4. Our mimicking upon one focal block is described in the following [Mimicking-block] algorithm:

**M2-1**: Simulate each of the four components of $\tilde{\varepsilon }\left[b|□\right]={\left({\tilde{\varepsilon }}_{b}^{\left(1\right)},{\tilde{\varepsilon }}_{b}^{\left(2\right)},{\tilde{\varepsilon }}_{b}^{\left(3\right)},{\tilde{\varepsilon }}_{b}^{\left(4\right)}\right)}^{T}$ from ${\tilde{H}}^{\left(h\right)}$ with h=1,…,4, individually.
**M2-2**: Solve the equation for ${\tilde{C}}_{b}$: $b=1,\ldots ,B_{□}$
  $$\tilde{\varepsilon }\left[b|□\right]=V_{□}{\tilde{C}}_{b}.$$
**M2-3**: All of the block-specific ensemble of *B* mimicries are subject to the 4D hypercubes’ boundary constraints and denotes the resultant ensemble of $B^{*}$ mimicries as $C_{□}^{*}={\left\{{\tilde{C}}_{b}={\left({\tilde{x}}_{b},{\tilde{u}}_{b},{\tilde{z}}_{b},{\tilde{w}}_{b}\right)}^{T}\right\}}_{b=1}^{B_{□}^{*}}$.

**Figure 11:**
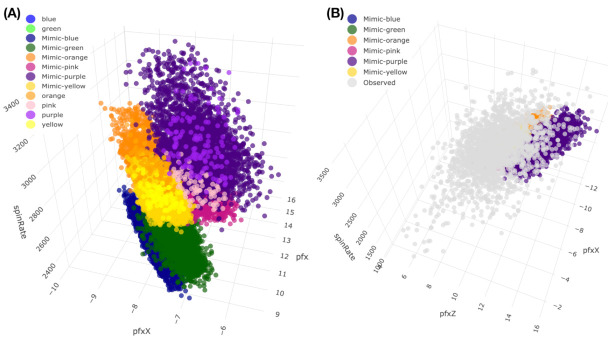
Snapshots of 3D plots of {“pfx_x”, “pfx_z”, “spin_rate”}: $C_{□}^{*}$ (dark-colored-points) and $C_{□}$ (light-colored-points) the six color-coded mimicked blocks: (**A**) local view; (**B**) global view. Block-specific color-coding: Yellow; Orange; Green; Blue; Pink; Purple. See the corresponding rotatable 3D plots through the link: https://rpubs.com/CEDA/Mimicking (accessed on 8 May 2021).

By using the above [Mimicking-block] algorithm, we can simulate $B_{□}^{*}$ copies that could be much larger than the size of the block *m*. From a geometrical perspective, we can visualize $C_{□}^{*}$ contrasting with $C_{□}$, the observed manifold within a focal block. As such, the six mimicked blocks are shown in panel (A) of Figure 11.

Via the local view, we achieve the coherent filling into each 4D hypercube within each block by mimicking. It is essential to keep in mind that such a mimicking-based filling is done in a fashion coherent with the data’s observed deterministic and stochastic structures. With the details being filled-in within each 4D hypercubes, the local view of each block reveals its evident characteristic structures. What impacts would such characteristic structures bring collectively?

The consequential answer is somehow surprising: global structural details, as shown in panel (B) of Figure 11 and see the corresponding rotatable 3D plot via the link in the legend of Figure 11. That is, the manifold’s “2D sheet” indeed consists of at least three layers. The mimicked six blocks collectively constitute a part of the middle layer. Such a demonstration without ambiguity is chiefly attributed to mimicking: the point-clouds right above and beneath the patch of unified mimicked blocks are spotted. What are the reasons behind such a composite structure of multiple thin layers? The answer to this question will mean very much regarding the Magnus effects in the PITCHf/x database.

**Figure 12:**
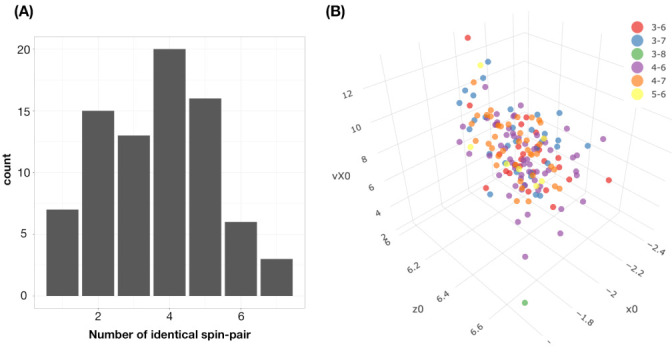
(**A**) Histogram of spin-pair (“spin_dir”, “spin_rate”) to (“pfx_x”, “pfx_z”) correspondence among 293 pieces of 4D hypercubes. (**B**) The snapshot of 3D plot of {“VX0”, “X0”, “Z0”} for data points belonging to five 4D hypercubes sharing the spin-pair (5,7). See the corresponding rotatable 3D plots through the link: https://rpubs.com/CEDA/Mimicking (accessed on 8 May 2021).

We investigate such causes by exploring the relational patterns that are embraced by the 293 pieces of 4D hypercubes contained in the contingency-4D-lattice shown in Figure 8. Because the Magnus effect is mainly regarding the force perpendicular to a spinning baseball trajectory, it is logical to see whether a single spin-pair of (“spin_dir”, “spin_rate”) indeed corresponds to multiple 4D hypercubes? It is unfortunate and fortunate at the same time that such multiplicity in correspondence is observed, as reported via a histogram in panel (A) of Figure 12.

Such an one-to-multiple correspondence means that one spin-pair, where one bin of “spin_dir” against one bin of “spin_rate” could have mapped to rather distinct multiple pfx-pairs of (“pfx_x”, “pfx_z”). This histogram concludes that the four features: {“pfx_x”, “pfx_z”, “spin_dir”, and “spin_rate”}, only provide an incomplete system description of baseball dynamics. This consequence simply implies that, even though the spin-pair (“spin_dir”, “spin_rate”) is a major factor of Magnus effect, it alone apparently can not reasonably lead to one single pfx-pair of (“pfx_x”, “pfx_z”).

Could a minor effect exist and help the predictive decision-making? To answer this question, it is natural to consider the biomechanics-related feature triplet: {“VX0”, “X0”, “Z0”} as potential individual minor factors or as a factor-group. Our explorations consist of making the following plots. Choose one spin-pair, say (5,7), for example. Its five corresponding 4D hypercubes are specified by five pfx-pairs of (“pfx_x”, “pfx_z”):{(3,6), (3.7), (4,6), (4,7), (5,6)}. The 3D rotatable plot of {“VX0”, “X0”, “Z0”} with respect to memberships of five 4D hypercubes also reveals a very high degree of mixing, as seen in panel (B) of Figure 12. Here, we conclude that neither individual features of {“VX0”, “X0”, “Z0”}, nor the triplet, play role of minor factors.

We then confirm that all features measured at pitcher’s mound: {“spin_dir”, “spin_rate”, “VX0”, “X0”, “Z0”}, can only incompletely prescribe pfx-pairs (“pfx_x”, “pfx_z”) in the aerodynamics of this particular pitcher’s four-seam fast-ball pitching dynamics. We recognize that such incompleteness renders no analytical systems of equations being possible for the pfx-pairs (“pfx_x”, “pfx_z”) as a 2D response variable with respect to the five covariate variables: {“spin_dir”, “spin_rate”, “VX0”, “X0”, “Z0”}. That is, based on this data set, any functional modeling upon the pfx-pair is unreasonable, at least from physics perspective. The generic algorithmic flow-chart of mimicking protocol for this scenario is described in Algorithm 4.

Here, we briefly summarize some of the key and significant conclusions from multiple perspectives. First, the foremost message is that Data Analysis must undertake mimicking the observed structural data matrix to figure out data’s authentic information content with structural dependency among all involving features. Secondly, we need to determine whether the computed information content indeed offers a complete or incomplete description of the system involving the targeted response variable. Thirdly, if completeness is confirmed, then our mimicking offers visible understanding of the targeted system and a model-free predictive decision-making platform. If any modeling structures are undertaken, then data’s structural dependency and multi-scale deterministic and stochastic parts of information content need to be accommodated. Consequential impacts on Machine Learning and Data Science are discussed in the last section. Fourthly, if the decision of incompleteness in describing the target system is reached, then this decision will postpone all attempts of modelling and predictive decision-making until extra features being discovered and measured.
**Algorithm 4:** Micmicking protocol for contingency-*k*D-lattice for scenario-II
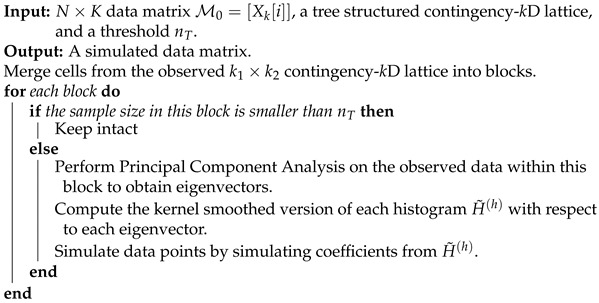


## 5. CEDA with Mimicking Scenario-III: Continuous and Categorical Features

So far, we have discussed mimicking under two pure scenarios: (1) all of the features being categorical; and, (2) all features being continuous, respectively. In this section, we discuss the mixed scenario: some features being continuous, and some are categorical. Again, we demonstrate that the mimicking protocol for this mixed scenario follows the same theme of mimicking the two pure scenarios. Therefore, the universal theme of mimicking a data matrix starts with categorical or categorized features in order to discover all of the large and median scale structural dependency among features by using contingency-*K*D-lattice, and then to capture heterogeneous fine-scale structural dependency within each *K*D hypercubes. For expositional simplicity, in this section, we consider mimicking a 2118×4 data matrix with four features: {“zone”, “VX0”, “X0”, “Z0”}. The techniques developed here would be easily expanded for mimicking the observed 2118×10 data matrix. The discussions of the impacts of mimicking this larger and complex data matrix would add many more pages to this already lengthy paper. Hence, the discussion will be reported in a separate work.

**Figure 13:**
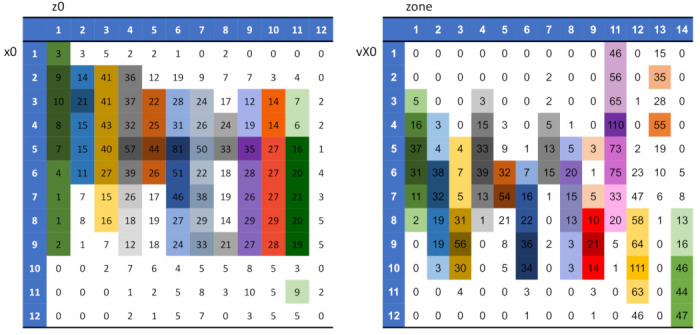
Two contingency tables of two feature-pairs: {“zone”,“VX0”} and {“X0”, “Z0”}. Observed column-wise ordering rules with 95% of reliability are marked. The collective acceptance rate is about 56% with respect to simply random sampling.

Based on the MCE matrix and directed network that are reported in Figure 2, we clearly see that these four features can subdivided into two feature-pairs: {“X0”, “Z0”} and {“zone”,“VX0”}. Accordingly, based on their histograms, we construct two contingency tables for these two pairs with 127 and 96 occupied cells, as shown in panels (A) and (B) of Figure 13, respectively. Upon these two contingency tables, as discussed in the two previous scenarios, we identify all of the column-wise ordering patterns and individually check whether their reliability reaches 95%. All of the confirmed ordering patterns with 95% reliability are taken as observed patterns and marked in these two contingency tables. These selected patterns are to be conditioned upon within all computations and operations for mimicking. That is, our mimicking protocol would accept a simulated a 2118×4 matrix {“X0”, “Z0”, “zone”, “VX0”} when all of these confirmed rules marked in panels (A) and (B) of Figure 13 are collectively satisfied. If the simple random sampling scheme is implemented upon observed data, we found that the acceptance rate is about 56%. That is to say that about 45% of bootstrapped data matrices fail to be accepted by the conditioning paradigm.

The contingency table of {“X0”, “Z0”} reveals unimodal patterns row- and column-wise. However, its overall 2D structural pattern seemingly is not Normal-like. As for the contingency table of {“zone”, “VX0”}, many interesting global patterns are observable beyond the column-wise unimodal patterns. Recall the strike-zone used by MLB, as shown in panel (A) of Figure 1. It is also noted that there is no zone-10. For zone-11 to zone-14 (the four columns on the right of the contingency table), we see that large cell-counts on zone-11 and zone-13 (on the left-hand side of outer peripheral zone coding) are associated with the first four rows with lower “vX0” values, while large cell-counts on zone-12 and zone-14 (on the right-hand side of outer peripheral of zone coding) are associated with the bottom four rows with high “vX0” values. Such a separation in speed (“vX0”) is also evidently seen within the strike-zone (zone-1 to zone-9). Lower speeds for zone-1, zone-4, and zone-7 (on the left-hand side of inner zone coding), while higher speeds for zone-3, zone-6, and zone-9 (on the right-hand side of inner of zone coding). Collectively, we see that this pitcher delivered higher speed pitches to the zones on the right than to the zones on the left.

Although we also see that “vX0” is informative enough to predict the left-hand-side against the right-hand side, it apparently cannot differentiate the upper-side against the lower-side of inner or peripheral zones. Accordingly, one natural question is: would {“X0”, “Z0”} help? This question can be specifically and precisely described as the following one: **If “VX0” is taken as a major factor for predicting “zone”, could the feature-pair {“X0”, “Z0”} play the role of a minor factor?**

**Figure 14:**
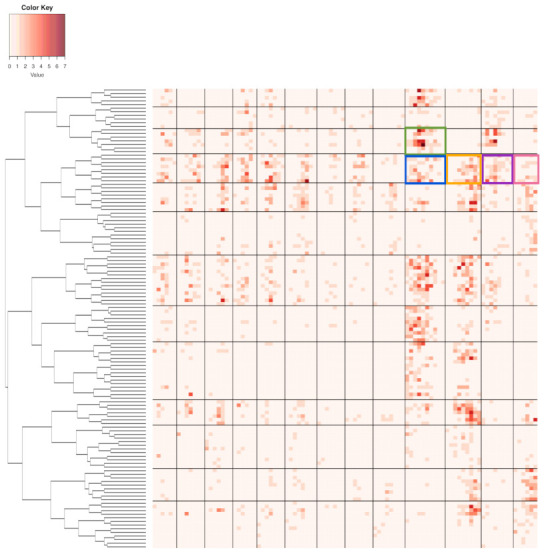
The 127×96 contingency-4D-lattice of two feature-pairs: {“X0”, “Z0”} vs. {“zone”,“VX0”}. The row-axis is superimposed with a HC-tree derived from 127×127 adjacency matrix under eight-neighbor-neighborhood system. These five blocks are marked across the four peripheral zones.

To address the above somehow equivalent questions, as discussed in the previous two scenarios, we build a 127×96 contingency-4D-lattice with 127 occupied cells of a contingency table of {“X0”, “Z0”} being arranged on its row-axis and 96 occupied cells of contingency table of {“zone”, “VX0”} on column-axis. Further, the row-axis of this 127×96 matrix-lattice is permuted with respect to an HC-tree derived based on the 127×127 symmetric binary adjacency matrix of the 127 cells under the eight-neighbor-neighborhood system. Because of the categorical nature of “zone”, the column-axis is arranged according to zone-numbers from left-to-right, and each zone-number is coupled with increasing bin-numbers of “VX0”. The heatmap resulted from the row, and column permutations are shown in Figure 14.

Indeed, this heatmap offers a platform that very clearly addresses the above questions. Interestingly, the answers are “yes” and “no” due to the heterogeneity of minor effects. We divide the whole heatmap lattice into a 13×13 lattice of blocks. Each block-column corresponds to one zone-number. By comparing the zone-12 block-column, which is the 3rd column from the right, and zone-14 block-column, which is the first column from the right, we can see that many blocks on the zone-14 column are empty or nearly empty, while blocks on the same rows on zone-12 block-column are heavily occupied. Although not as many, the reverse correspondences are also seen. Such pairs of having a heavily-occupied block against an empty block on either zone-12 or zone-14 sharing the same block-row strongly indicate that feature-pair {“X0”, “Z0”} can help to separate zone-12 and zone-14. These pairs are “yes” answers. Accordingly, “yes” pairs can be found nine out of 13 block-rows. However, the {4,5,12,13} block-rows offer visible “no” pairs. Similar but less clear patterns are visible by comparing block pairs on zone-11 and zone-13 block-columns across all block-rows. In sharp contrast, when comparing zone-1 through zone-9 block-columns, the nine blocks on each block-rows are equally occupied. Therefore, we conclude that feature-pair {“X0”, “Z0”} can help to differentiate zone-12-against-zone-14 and zone-11-against-zone-13 to a great extent, but have zero differentiability for zone-1, 2, 3-against-zone-7,8,9. Such visible and explainable heterogeneity in high order structural dependency is the typical phenomenal manifestation of a minor-factor.

In summary, the global and large scales patterns of structural dependency found in the two contingency tables in Figure 13 and in the heatmap of contingency-4D-lattice, as shown in Figure 14, collectively constitute key information content contained in 2118×4 data matrix with four features: {“zone”, “VX0”, “X0”, “Z0”}. Based on such structural dependency patterns, mimicking can and must be carried out accordingly. Consequently, the resultant mimicked data will contribute not only fine-scale details, but also enhanced block-by-block geometric structures that are only vaguely visible based on observed data.

Our mimicking protocol that is given below for such data matrix of mixed data types bears the same theme used in the previous sections.

[Mimicking protocol for a matrix of mixed data types:]

We take the heatmap of contingency-4D-lattice that is shown in Figure 14 as a road map of our mimicking since it contains all patterns of large scale structural dependency.

**M3-1**. Simulate a contingency table of {“zone”,“VX0”} with 96 occupied cell-counts that satisfy all confirmed rules marked in panel (B) of Figure 13;
**M3-2**. We build a HC-tree based on the 127×127 binary adjacency matrix of the occupied cells in the contingency table, as shown in panel (A) of Figure 13. Because of the categorical nature of “zone”, we do not have 96×96 adjacency matrix for the 96 occupied cells in panel (B) of Figure 13. We build 127×96 contingency-4D-lattice with the HC-tree being superimposed on its row-axis. After some explorations, we choose 13 branches of this HC-tree as 13 clusters of 2D cells of {“X0”, “Z0”}. The 96 cells are arranged with respect to increasing zone-numbers, within each of which increasing bin-numbers are arranged on its column-axis. These 96 2D cells of {“zone”,“VX0”} are divided in 13 with respect to their zone-numbers, respectively. Consequently, one 13×13 block-lattice is built upon the 127×96 contingency-4D-lattice. Here, each block is one zone-number specific.
**M3-3**. Based on the simulated 96 cell-counts, we simulate cell-counts of 127×96 matrix in a column-by-column fashion. The 127 row-sums must satisfy the confirmed rules marked in contingency table of {“X0”, “Z0”}, as shown in panel (A) of Figure 13.
**M3-4**. Within each block, we simulate continuously measured {“VX0”, “X0”, “Z0”} data points via PCA and the kernel smoothing methodologies with the imposed boundary constraints of all involving 3D cubes within the block, as described in the previous section for the setting of all features being continuous. Such block-by-block mimicking affords us to have as many simulated data points as we wish to have.
**M3-5**. Among the mimicked data points within each zone-number specific 3D cubes derived in Step-M3-4, we use the simple random sampling scheme to sample the simulated counts (in Step-M3-3) of mimicked data points. Collectively, we build a mimicked 2118×4 data matrix of four features: {“zone”, “VX0”, “X0”, “Z0”}. We can have as many copies of such mimicry as we wish to have.

Algorithm 5 describes the generic algorithmic flow-chart of mimicking protocol for this scenario.
**Algorithm 5:** Micmicking protocol for contingency-*k*D-lattice for scenario-III**Input**: N×K data matrix $M_{0}=\left[X_{k}\left[i\right]\right]$, a tree structured contingency-*k*D lattice, and a threshold $n_{T}$.
**Output**: A simulated data matrix.
Simulate cell counts by Algorithm 3 with rules set for all categorical variables. Simulate variable values by Algorithm 4 within each obtained blocks with size larger than $n_{T}$ for all continuous variables. 


There are five heavily occupied blocks marked in Figure 14 with five different color-codings. They are located on two adjacent block-rows (one in the 3rd block-row and four in the 4th block-rows) and four adjacency block-columns (two in zone-11 block-column, and one in each of zone-12, zone-13, and zone-14). Two block-pairs are established: (zone-12, zone-14) and (zone-11, zone-13), on the 4th block-row. They both give rise to a “no” answer. We perform mimicking upon the five blocks separately.

**Figure 15:**
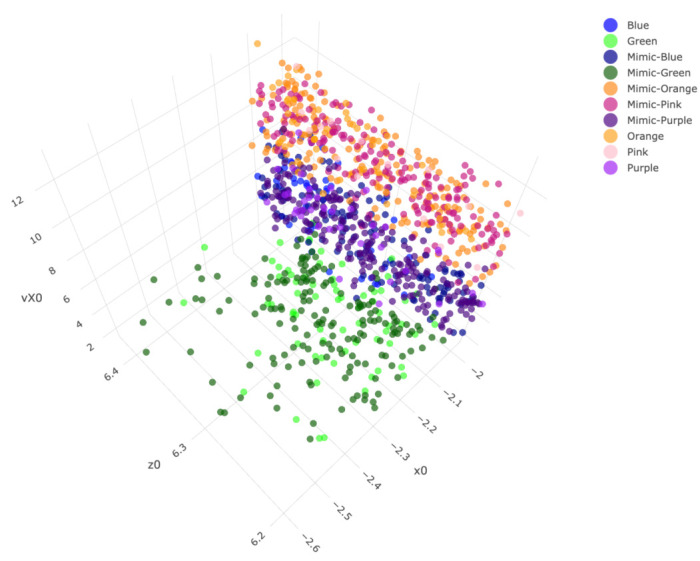
Geometry of five mimicked blocks marked on the heatmap of 127×96 contingency-4D-lattice of two feature-pairs: {“X0”, “Z0”} vs. {“zone”,“VX0”}, as shown in Figure 14. These 5 blocks are marked across the 4 peripheral zones. See corresponding rotatable 3D plots through the link: https://rpubs.com/CEDA/Mimicking (accessed on 8 May 2021).

In Figure 15, we demonstrate the geometric display of the mimicked data of the five blocks marked in Figure 14 in the form of three 3D bricks. Two of three 3D bricks are for the two block-pairs. Within each brick, the observed and mimicked data points are very well mixed. The 3D geometry of the three 3D bricks reflects the neighboring relations of their two block-rows (3rd and 4th) and four block-columns (zone-11 to zone-14) within the heatmap display very well, as shown in Figure 14. The geometry of three 3D bricks also reflects the row difference. The green-color-coded brick is by itself because it is the only block on the 3-row. In contrast, the row-specific 4-column neighboring relations are functionally reflected by merging and mixing within each block-pairs in the geometry of 3D bricks. Such merging and mixing within each of the two block-pairs is seen as being functional. Mimicking makes such geometric and functional revelations visible and explainable.

It is also essential to keep in mind that the adjacency-matrix-based HC-tree makes such geometric and functional relations found on and between the geometry of 3D bricks and neighboring system of blocks in contingency-4D-lattice possible. This recognition would play a key role when we undertake mimicking the observed 2118×10 involving with three synergistic feature-groups: {“zone-number”, “batting-result”, “Ball-vs.-Strike” }, {“pfx_x”, “spin_dir”, “pfx_z”, “spin_rate”}, and {“x0”, “z0”, “VX0”} in a separate study.

Indeed, our computing endeavors in this setting of mixed data types illustrate the chief message of this paper: by building and revealing data’s categorical nature, mimicking-based data visualizations will lead to a natural platform for exploratory data analysis (EDA). In the next section, we conclude and then briefly discuss the impacts of our Categorical Exploratory Data Analysis (CEDA) with regard to mimicking on Machine Learning and Statistics and Data Science.

## 6. Conclusions: Impacts of CEDA with Mimicking on ML, AI and Statistics

A structured data matrix is, in general, created to describe a system of interest. A set of features is primarily chosen based on data curators’ domain knowledge and subject matter expertise. In this paper, we point out the possibility that such a set of features might only provide an incomplete description of the target system. Beyond speculating such possibility intuitively, we actually demonstrate how to see it in a visible and explainable manner. Consequently, we conclude that the ultimate goal of data analysis upon a data matrix is, and should be, phrased as discovering data’s authentic information content in full.

In this paper, we build a computational paradigm, called CEDA, with mimicking to discover such information content. CEDA discovers the global and large scales of information content in terms of deterministic and stochastic structures that have to be extracted from all features’ categorical nature, despite their data types: continuous, discrete, or categorical. That is, both scales of structures are typically categorical. Such categorical nature facilitates high potentials to accommodate non-linear and complex relational dependency among features. This key part of data analysis is, by-and-large, missing in the literature.

Further, patterns of categorical multiscale structural dependency collectively serve as the basis for mimicking. After securely preserving data’s global and large scales structures, mimicking is further designed to extract fine-scale dependency in the fashion of one-locality-at-time. Such a locality-by-locality design allows the mimicking to expose data’s fine-scale information heterogeneity. By design, all of the mimicries are supposed to retain all scales of computed characteristics belonging to an observed data matrix. In this fashion, our computing endeavors must discover data’s information content first. Above all its potential merits, such information content primarily offers a visible and explainable understanding of the system of interest. It also provides a natural platform for us to differentiate which system descriptions are complete and incomplete. Afterward, we can afford all of the relevant tasks of inference and reliability related evaluations.

For example, based on categorical patterns revealed in the contingency tables presented in Figure 13 and the heatmap displayed in Figure 14, we are very confident that the four features’: {“zone”, “VX0”, “X0”, “Z0”} description of the pitching system pertaining to this pitcher’s 4-seam Fastball is incomplete in the sense of being unable to differentiate the 13 zones individually with respect to biomechanics via {“VX0”, “X0”, “Z0”}. This incompleteness is marked by the fact that 12 out of 13 individual zone-numbers are not identifiable. However, it is somehow complete from the perspective of the Left-vs.-Right and peripheral-vs.-inner parts of the strike zone.

Another example of incomplete system description is about the aerodynamic system of Magnus effects via {“pfx_x”, “spin_dir”, “pfx_z”, “spin_rate”}. The incompleteness is well revealed in a fashion of one single spin-pair corresponding to multiple pfx-pairs that are reported in Figure 12. That is, the majority of spin-pairs bear no predictability of pfx-pairs.

Such incompleteness of a system description will exclude any meaningful modelings upon a set of fechosenatures. Checking whether a set of features completely describes a targeted systemis not a simple task. The complexity pertaining to this task can be clearly defined as discovering which features play the major roles, which features play only minor roles, and which features play no roles at all, as we have demonstrated in this paper. Indeed, such major-vs.-minor effects naturally reflect the multiscale heterogeneity embraced by data’s information content. Hence, once again, the computing task for data’s full information content is not only a critical topic for statistics and data analysis, but equally critical for Machine learning (ML) and Artificial Intelligence (AI).

Nevertheless, this critical topic seemingly has not yet attracted serious research attention in all related literatures. It is partly because any assumed modeling structures likely violate certain visible and explainable data characteristics. Furthermore, it is partly, because, even when the completeness of a system description is confirmed, any valid model needs to accommodate all computed multiscale deterministic and stochastic structures. Such a valid model might be too complicated to be realized because of the complexity of its multiscale nature. From both perspectives, we explain why all models are wrong. Are some models more useful than others? Our answer to this somehow self-contained question is: as the data’s information content is computable, why are any ad hoc choices of models of any realistic uses? We believe that such an answer bears awakening impacts in the fields of Statistics, Data Science, ML, and AI.

In Statistics, a choice of modeling structure usually specifies explicit parameterized functional relations between response and covariate variables coupled with an additive error variable. By placing model structures upon an observed data matrix and skipping the task of figuring out data’s information content, statistical analysis goals are focused on estimating the parameters and specifying one seemingly valid distribution among members of an assumed family. The principles and approaches available for achieving these goals have become standard receipts. For instance, the approaches for goodness-of-fit testing on modeling structures often lead to single testing statistics, which usually aims at only whether one selected structural characteristic of the modeling is present in the data or not. The maximum likelihood estimating (MLE) principle also leads to a single estimating statistics. Such kinds of single real-valued statistics only offer limited amounts of information. Further, these statistics’ variations and reliability evaluations are typically specified by asymptotical Gaussian approximations or Bootstrapping via simple random sampling under the i.i.d framework. Such receipt-like analysis has been adopted by many scientists far beyond statistics and computer science. At the current state of data analysis, such model-based receipt-like analysis has been widely mistaken and wrongly accepted as “what data analysis is all about”.

Here, our standpoint toward the above state of data analysis is precisely given, as follows. CEDA with mimicking apparently must be performed before implementing the above rigid statistical model-based data analysis. Even if any completeness of system descriptions are found being supported by data, statistical modeling could be just too difficult, if not entirely hopeless, to be properly constructed in real-world settings due to complexity involving accommodating the multiscale deterministic and stochastic structures. Even when a modeling structure is confirmed to be valid, the reliability evaluations that are based on an ensemble of mimicries of observed data matrices could be very distinct to that-based bootstrapping. This potential is real, since very high percentages of the simple random sampling-based bootstrapped data matrix are rejected in our mimicking protocols throughout the three settings discussed in this paper. Further, classic reliability evaluations are performed by slightly perturbing deterministic as well as stochastic structures. Such a perturbation scheme likely is not in accordance with reality, because these two kinds of structures indeed interact and produce structural changes in the real world. On top of structural interactions, there are too many potential perturbation schemes due to multiple scales structures in a data matrix. In sharp contrast, an ensemble of mimicries of observed data matrix enable us to realize reliability evaluations of any computed patterns being discovered through CEDA. Such a reliability concept primarily and purely rests on data’s stochasticity, not a man-made one.

Nowadays, predictive inference has nearly become the solo goal of so-called data science performed, in particular, by computer scientists. ML approaches, such as Random Forest and many Boosting variant approaches, are driving the predictive inferences toward achieving the “engineering-like of precision” by enlarging their underlying black-boxes. Such ML approaches commonly developed by dividing a data set into two subsets: testing and training. Beneath all attempts to achieve the universal goal of minimizing the prediction error rate as much as possible; nevertheless, there is one serious but unspoken requirement that needs to be fulfilled underlying all ML applications. This requirement is that both testing and training data subsets are identical regarding all aspects of data structures in the original data set.

The task of checking whether this requirement is fulfilled or not means that the multiscale information content in these two data subsets, or data submatrices, must be computed and discovered, respectively. Additionally, methodologies need to be developed for checking the information content equivalence. In other words, CEDA with mimicking has to be performed twice before checking the structural equivalence requirement. However, such a requirement is hardly being checked in real-world ML applications or within Artificial Intelligence (AI) products. The consequences of without checking this requirement surely are linked to social injustices, because the differences between the testing data subset and training data subsets would make the error-rate evaluations invalid. More seriously, and importantly, the structural distinctions between the original data set and the populations, which use Artificial Intelligence (AI) products, are multiple avenues for creating social injustices.

In summary, our CEDA with mimicking can help to develop ML research and build AI products that achieve the requirement for eliminating the potentials of social injustices. Additionally, CEDA with mimicking would bring out the realistic data analysis in the real world and, at the same time, serve as one fundamental bedrock of Data Science.

## Data Availability

The pitching data are available in PITCHf/x database belonging to Major League Baseball via http://gd2.mlb.com/components/game/mlb (accessed on 8 May 2021).

## References

[1] Steinbeck J. (1951). The chapter of March, 20, Easter. The Log From The Sea of Cortez.

[2] Anderson P.W. (1972). More is different. Science.

[3] Donoho D.L. (2017). 50 years of data science. J. Comput. Graph. Stat..

[4] Gelman A. (2003). A Bayesian formulation of exploratory data analysis and goodness-of-fit testing. Int. Stat. Rev..

[5] Gelman A., Vehtari A. (2021). What are the most important statistical ideas of the past 50 years?. arXiv.

[6] Tukey J.W. (1962). The future of data analysis. Ann. Math. Statist..

[7] Briggs L. (1959). Effect of Spin and Speed on the Lateral Deflection (Curve) of a Baseball and the Magnus Effect for Smooth Spheres. Am. J. Phys..

[8] Fushing H., Roy T. (2018). Complexity of Possibly-gapped Histogram and Analysis of Histogram (ANOHT). R. Socity Open Sci..

[9] Fushing H., Liu S.-Y., Hsieh Y.-C., McCowan B. (2018). From patterned response dependency to structured covariate dependency: Categorical-pattern-matching. PLoS ONE.

[10] Cox D.R., Hinkley D.V. (1974). Theoretical Statistics.

[11] Tufte E.R. (1983). The Visual Display of Quantitative Information.

[12] Wilkinson L. (2005). The Grammar of Graphics.

[13] Li M., Vitanyi P.M.B. (2009). An Introduction to Kolmogorov Complexity and Its Applications.

[14] Chou E.P.-T., McVey C., Hsieh Y.-C., Enriquez S., Fushing H. (2020). Extreme-K categorical samples problem. arXiv.

[15] Girvan M., Newman M.E.J. (2002). Community structure in social and biological networks. Proc. Natl. Acad. Sci. USA..

[16] Chen C., Fushing H. (2012). Multi-scale community geometry in network and its application. Phys. Rev. E.

[17] Fushing H., Chou E.P. (2020). Categorical Exploratory Data Analysis: From Multiclass Classification and Response Manifold Analytics perspectives of baseball pitching dynamics. arXiv.

[18] Efron B. (1979). Bootstrap methods: Another look at the jackknife. Ann. Statist..

